# A dataset of transcriptomic effects of camptothecin treatment on early zebrafish embryos

**DOI:** 10.1016/j.dib.2024.111041

**Published:** 2024-10-16

**Authors:** Sergey V. Prykhozhij, Kevin Ban, Zane L. Brown, Kim Kobar, Gabriel Wajnberg, Charlotte Fuller, Simi Chacko, Jacynthe Lacroix, Nicolas Crapoulet, Craig Midgen, Adam Shlien, David Malkin, Jason N. Berman

**Affiliations:** a Children's Hospital of Eastern Ontario (CHEO) Research Institute and University of Ottawa, Ottawa, ON, Canada; b Dalhousie University Medical School, Halifax, NS, Canada; c Atlantic Cancer Research Institute, Pavillon Hôtel-Dieu, Moncton, NB, Canada; d HHS McMaster University Medical Centre, Division of Medical Microbiology, Hamilton, ON, Canada; e Department of Pathology, Dalhousie University, Halifax, NS, Canada; f IWK Health Centre, Halifax, NS, Canada; g Genetics and Genome Biology Program, Division of Hematology/Oncology, The Hospital for Sick Children, PGCRL, Toronto, ON, Canada; h Departments of Pediatrics and Medical Biophysics, University of Toronto, Toronto, ON, Canada

**Keywords:** Zebrafish (Danio rerio), Camptothecin, Topoisomerase I inhibitor, p53, Interferon-stimulated gene, Chemotherapy

## Abstract

Zebrafish (*Danio rerio*) are a good model for cancer research including studies on chemotherapy treatments. We treated wild-type and *miR-34a* deletion mutant zebrafish embryos at 24 h post-fertilization with 1 µM of the topoisomerase I inhibitor, camptothecin (CPT), for 4 h to catalogue gene expression changes induced by this DNA damage treatment and to understand if these changes are influenced by loss of miR-34a. The 4 sample groups of 3 independent biological samples consisting of 30 embryos each were analyzed by RNA-sequencing using the recently updated zebrafish transcriptome annotation based on GRCz11, which enabled a more complete and sensitive read mapping and gene assignment than standard annotations. Using this gene expression estimates dataset as the primary resource, we performed a differentially expressed gene (DEG) analysis based on treatment as loss of miR-34a had minimal effects on CPT-induced expression changes. The DEGs were analyzed for Gene Ontology and KEGG pathway terms. Enriched terms and pathways among up-regulated genes were mostly related to stress, cell death, cell cycle regulation, transcriptional regulation, cell signalling, developmental processes and synthesis of retinol and steroid hormones. By contrast, down-regulated genes were most strongly associated with genes involved in key developmental processes, adhesion molecules, as well as some transport and metabolic pathways, together suggesting a “developmental shutdown”. We also identified interferon-regulated genes and p53 target genes activated or inhibited by DNA damage due to topoisomerase I inhibition, suggesting that they are important components of the response to this type of DNA damage in zebrafish embryos.

Specifications Table

**Table:** 

Subject	Biological Sciences; Omics: Transcriptomics; Cancer Research; Developmental Biology
Specific subject area	Transcriptomics for developmental toxicology of topoisomerase inhibitors; zebrafish animal models for studies of p53 biology and cancer research
Data format	Raw Analyzed Filtered
Type of data	Table Chart Graph Figure
Data collection	Wild-type and *miR-34a-/-* embryos at 24 hpf were divided into triplicate control and treatment groups (each of 30 embryos) and treated with either 0.005 % DMSO or 1 µM CPT in E3 fish medium for 4 h followed by RNA extraction, sequencing and analysis as described in Materials and Methods.
Data source location	Halifax, Nova Scotia, Canada – initial sample preparation Moncton, New Brunswick, Canada – RNA sequencing
Data accessibility	The processed and analyzed data are deposited with the article. The original raw data was deposited in Gene Expression Omnibus (GEO) under the identification number GSE142440. Direct URL to data: https://ncbi.nlm.nih.gov/geo/query/acc.cgi?acc=GSE142440
Related research article	miR-34a is a tumor suppressor in zebrafish and its expression levels impact metabolism, hematopoiesis and DNA damage. Prykhozhij SV, Ban K, Brown ZL, Kobar K, Wajnberg G, Fuller C, Chacko S, Lacroix J, Crapoulet N, Midgen C, Shlien A, Malkin D, Berman JN. PLoS Genet. 2024 May 28;20(5):e1011290. doi: 10.1371/journal.pgen.1011290. [1]

## 1 Value of the Data

• This dataset provides a transcriptome-level description of how zebrafish embryonic development is affected by DNA damage due to treatment with camptothecin, a topoisomerase I inhibitor.
• DNA damage treatments are used by many zebrafish animal model researchers to test fundamental and applied hypotheses. However, there is limited transcriptomic data on the response of zebrafish embryos to this type of DNA damage. Our dataset can thus inform researchers using DNA damaging treatments about the zebrafish embryo responses to them.
• This dataset enables queries of whether individual genes are regulated by camptothecin-induced DNA damage as well as testing if certain gene sets are over-represented in this dataset.

## 2 Background

Our initial motivation for this study was to understand whether the transcriptomic response to p53 activation can be affected by the loss of microRNA-34a in zebrafish. Human *MIR34A* was the first microRNA gene identified as a p53 target, which is consistent with evidence that miR-34a overexpression stimulates apoptosis and cell cycle arrest [[2], [3], [4], [5]]. Several studies also reported increased tumor development in oncogene-expressing or tumor suppressor mutant mice when MiR-34a or all 3 miR-34s are lost [6,7]. To induce DNA damage and the associated transcriptomic response we used camptothecin (CPT), a DNA damage-inducing topoisomerase I inhibitor [8], which activates p53 in zebrafish [9] and whose analogues are used extensively as chemotherapeutic agents [10]. Although there was no transcriptomic data on CPT treatment in zebrafish embryos, we were aware that CPT is known to induce the cyclic GMP-AMP synthase - Stimulator of Interferon Genes (cGAS-STING) pathway in multiple cellular contexts [11]. We therefore aimed to obtain the overall data on the transcriptomic response to CPT treatment and assess whether *miR-34a* loss influences this response or at least subsets of it. All experiments were conducted in the casper mutant (*mitfa*^w2/w2^; *mpv17*^a9/a9^) background [12] as the wildtype. Neither of the mutations in this strain is known to influence the response to DNA damage or CPT treatment in particular.

## 3 Data Description

**Fig. 1:**
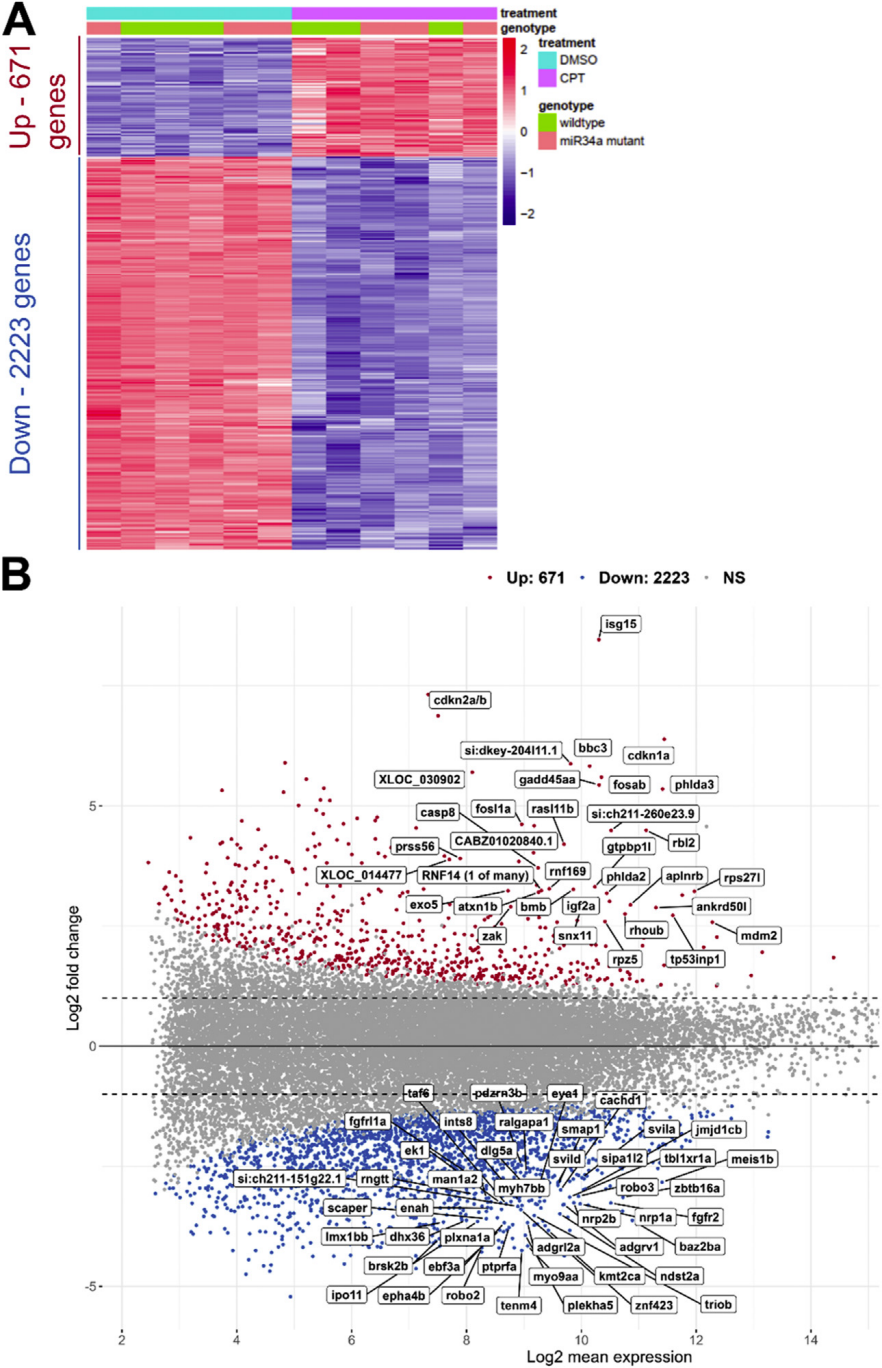
Global overview of differential gene expression after camptothecin (CPT) treatment of zebrafish embryos. **(A)** Heatmap plot of all DEGs after CPT treatment. Standard hierarchical clustering was performed of the variance-stabilized values which were scaled row-wise for visualization. Treatment and genotype factors are indicated in the heatmap. Numbers of up- and down-regulated genes are indicated in the side panel of the heatmap. **(B)** MA plot of all genes with non-DEG genes labeled in grey, up-regulated genes in red and down-regulated in blue. The names of top 80 DEGs are indicated directly on the plot. (For interpretation of the references to color in this figure legend, the reader is referred to the web version of this article.)

In this study, we are reporting expression changes and associated functional term enrichments in the transparent (except eyes) *casper* double mutant [13] (used as wild-type here) and *miR-34a-/- casper* embryos at 28 hpf after their 4-h treatment with DMSO (0.005 %) or 1 µM CPT in fish medium. We previously determined that loss of *miR-34a* does not significantly influence the CPT-induced expression changes [1] and we have therefore combined all data in a single count matrix and focused the differential expression analysis on the treatment factor alone. We also used a new transcriptome annotation [14] to improve the sensitivity of detecting relevant regulated genes. The resulting increase in the number of samples and the new annotation will likely improve sensitivity of detecting differentially expressed genes (DEGs) due to CPT and we used DESeq2 to ensure a high level of stringency to better detect enrichment of functional terms. With these settings, we identified 2894 such DEGs, among which there were 2223 down-regulated genes and 671 up-regulated genes (Fig. 1A). The MA plot (M - log ratio and A - mean average) shows the symbols of top 80 DEGs to provide a snapshot of the genes the expression of which was affected by CPT treatment (Fig. 1B).

**Fig. 2:**
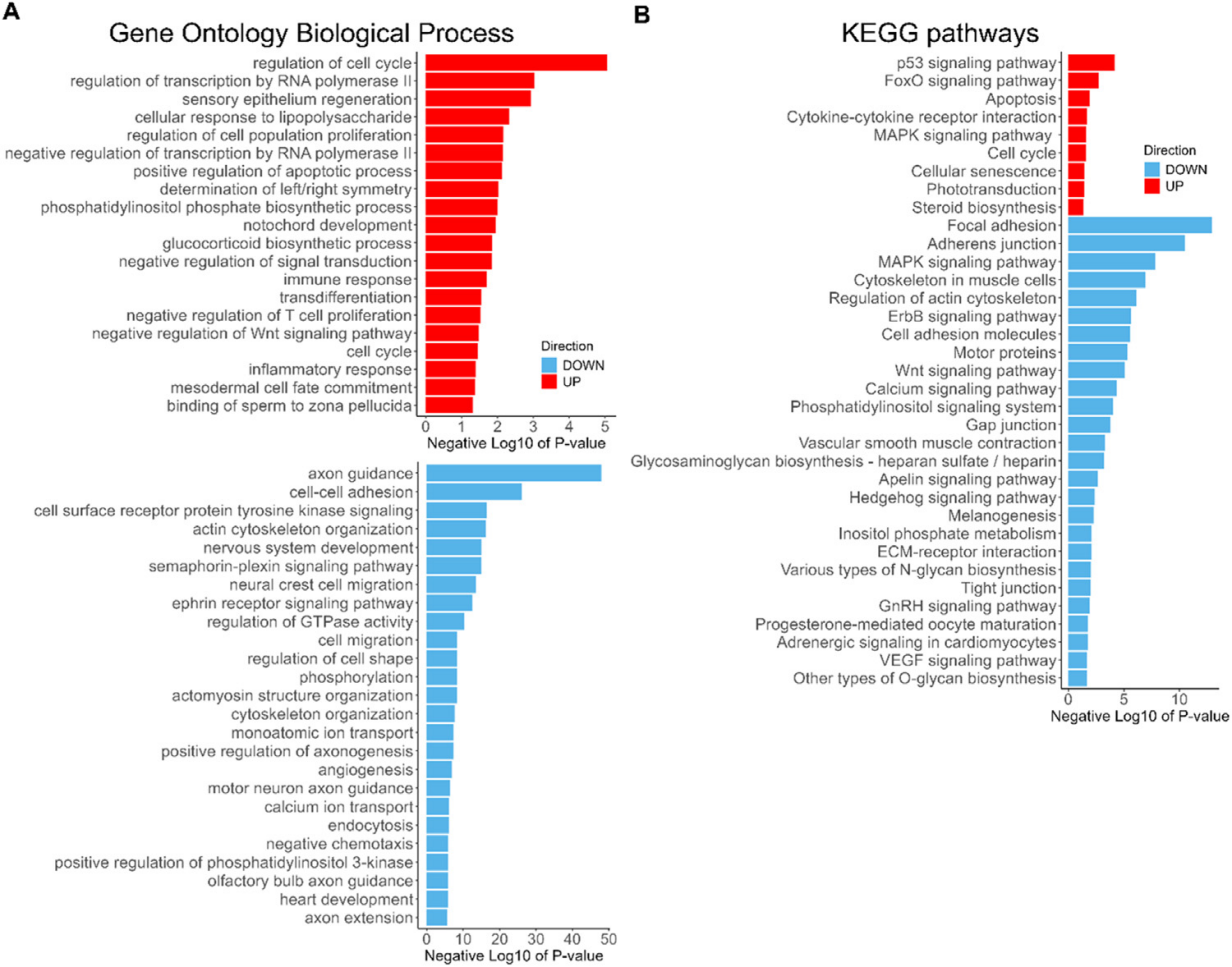
GO BP and KEGG Pathways terms enriched among the DEGs due to CPT treatment. Barplots of the Negative Log10 transformation of *P*-values for Gene Ontology Biological Process **(A)** and KEGG pathway **(B)** terms. **(A)** All of the GO BP terms associated with up-regulated genes (shown in red) are plotted as well as top 25 GO BP terms linked to down-regulated genes (light-blue). Separate plots are due to different scales of Negative Log10 of P-value. **(B)** KEGG pathway term plot. Direction of gene regulation is indicated by the legend on both sets of plots. (For interpretation of the references to color in this figure legend, the reader is referred to the web version of this article.)

The combined dataset DEGs were next analyzed for enriched Gene Ontology Biological Process (GO BP) terms and Kyoto Encyclopedia of Genes and Genomes (KEGG) pathways. For up-regulated genes, the most enriched terms for both GO BP and KEGG pathways were related to cell cycle regulation and apoptosis as underscored by enrichment of the ‘p53 signaling pathway’ (Supplementary Data; Fig. 2). Transcriptional regulators such as ‘FoxO signaling pathway’ and transcription-inducing signaling pathways such as ‘MAPK signaling’ were frequently up-regulated, whereas some other pathways were negatively regulated by the up-regulated genes, e.g. ‘negative regulation of signal transduction’ term includes several inhibitors of the JAK-STAT signaling pathway, and ‘negative regulation of Wnt signaling pathway’ was also enriched. Several immunity and inflammation-related terms were also enriched among up-regulated genes (Supplementary Data; Fig. 2).

The genes down-regulated by CPT treatment were more abundant in our dataset and the enriched GO BP and KEGG pathway terms were numerous and highly significant. Enriched among down-regulated genes were multiple signaling pathways such as ErbB, ephrin, semaphorin-plexin, Hedgehog, Wnt, MAPK, VEGF, GnRH, Insulin, calcium signaling, Apelin signaling, small GTPase-mediated signal transduction and the phosphatidylinositol signaling system. Cell adhesion machinery genes, including adherens junctions, gap junctions and tight junctions were also strongly down-regulated (Supplementary Data; Fig. 2). Migration-related and cytoskeletal terms were likewise enriched among the down-regulated genes such as neural crest cell migration, neuron migration, actin cytoskeleton organization, actomyosin structure organization, microtubule-based movement, positive regulation of filopodium assembly, cytoskeleton-dependent intracellular transport, and substrate adhesion-dependent cell spreading. Gene expression for multiple neural functions was strongly affected, such as the top down-regulated term ‘axon guidance’ and related terms: regulation of cell shape, positive regulation of axonogenesis, motor neuron axon guidance, negative chemotaxis and axon extension. Thus, most neural and other organ system developmental processes were strongly down-regulated, such as nervous system development, heart development, embryonic viscerocranium morphogenesis, somitogenesis, muscle cell development, angiogenesis, vasculature development, skeletal system development, cartilage morphogenesis and glomerulus development (Supplementary Data; Fig. 2). These terms and pathways enriched among DEGs provide a detailed picture of the zebrafish embryo response to DNA damage, which broadly involves up-regulation of multiple pathways necessary to respond to stress and an extensive shut-down of active developmental processes.

**Fig. 3:**
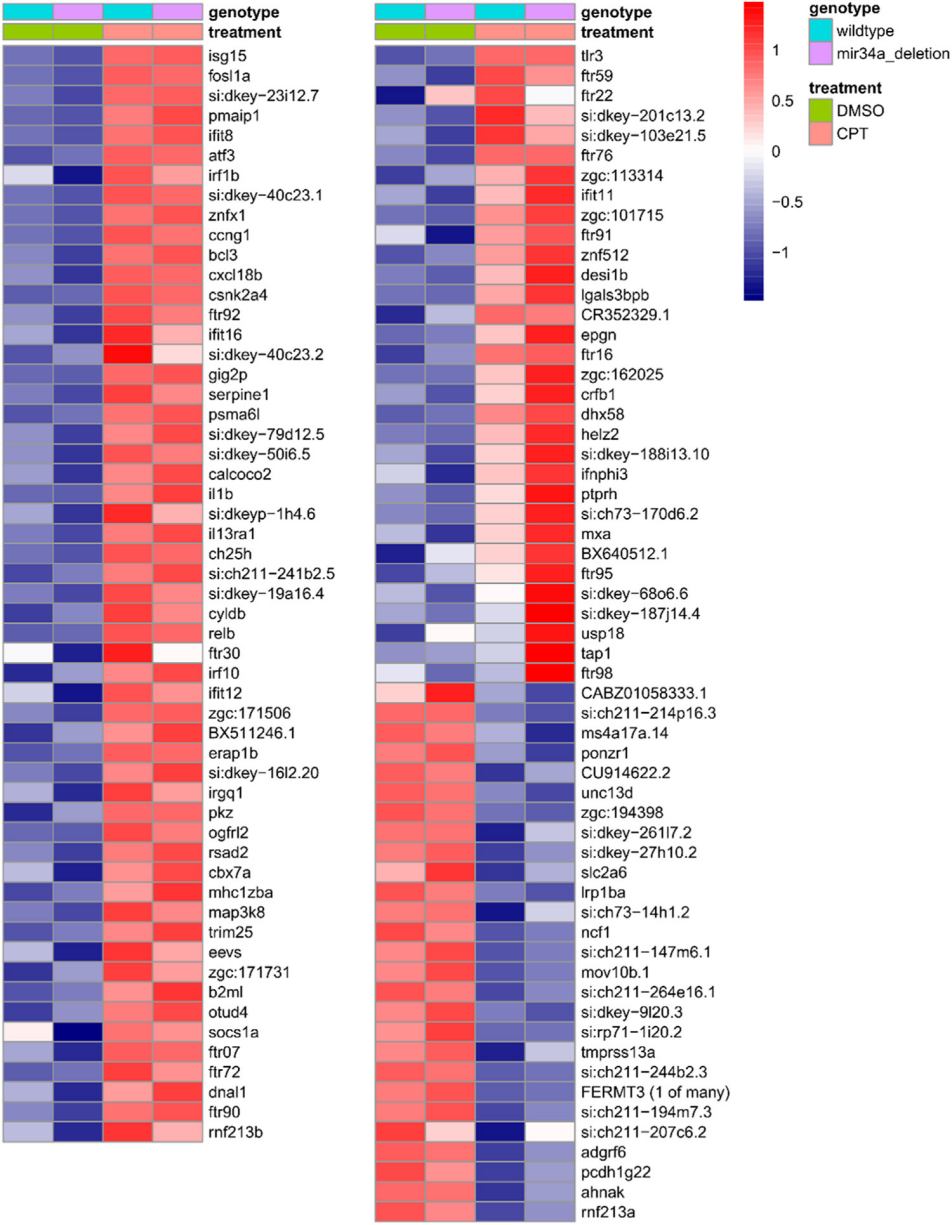
Heatmap of expression values for interferon-regulated genes (IRG) induced or repressed by DNA damage due to camptothecin treatment. Heatmap of normalized variance-stabilized gene expression values for known IRGs. To simplify presentation, averages of 3 biological replicates belonging to each treatment group are shown in the heatmap. Both up and down-regulated IRGs sorted in descending order by fold change are shown in the heatmap. The values were scaled row-wise. Treatment and genotype assignments are indicated by color bars above the heatmap, and the legend is provided at the top right.

Given that the top up-regulated gene in our dataset is *isg15*, we reasoned that our dataset may help us detect expression changes in multiple interferon-regulated genes (IRG). DNA damage is known to activate cGAS-STING pathway that detects cytosolic double-stranded DNA and activates the type I interferon (IFN) response [15]. We explored IRG induction, a hallmark of cGAS-STING pathway activation, using datasets from a study on IRGs in zebrafish [16], among which most were up-regulated IRGs and others were repressed by interferon-inducing stimuli. We compiled all IRGs from multiple experiments in that study and came up with 641 known zebrafish IRGs (Supplementary Data; “IFN-regulated genes” sheet). To determine the presence of IRGs more sensitively, we applied edgeR library to the count matrix with the same settings (absolute fold change ≥ 2; P-value ≤0.05) as in the related research article [1] and identified 114 significant ISGs (87 up-regulated and 27 down-regulated) (Fig. 3), making up 26.7 % of all known IRGs in zebrafish whose expression was detectable in the dataset (429 genes) (Supplementary Data; “IFN-regulated genes” sheet). Importantly, type I IFN ligand genes, *ifnphi3* and *il1b*, were induced, associated with innate immunity cGAS-STING pathway activation by DNA damage in zebrafish embryos [17].

**Fig. 4:**
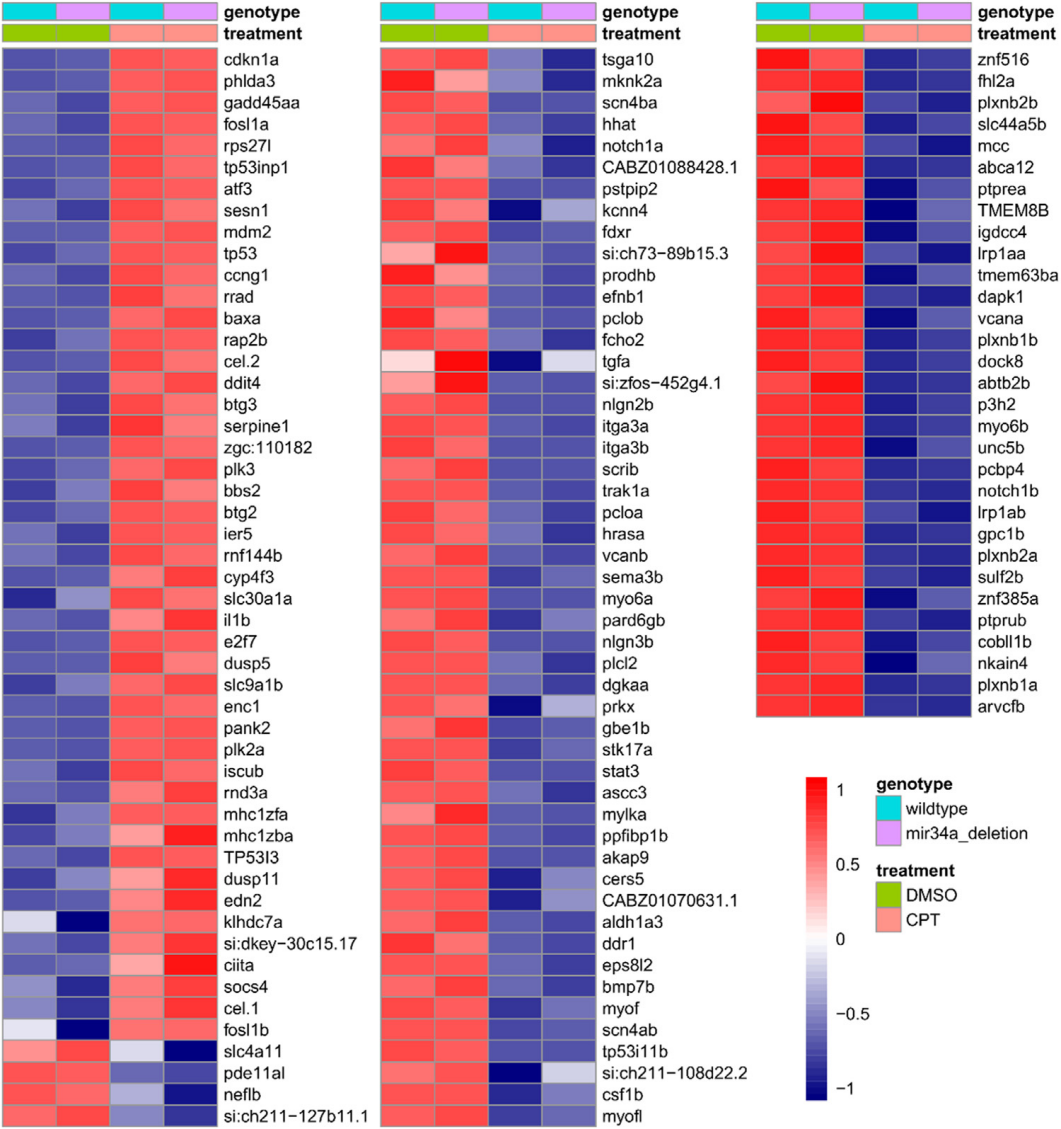
Heatmap of expression values for p53 target genes induced or repressed by DNA damage due to camptothecin treatment. Heatmap of normalized variance-stabilized gene expression values for known zebrafish orthologs of known human p53 target genes. Averages of 3 biological replicates belonging to each treatment group are shown in the heatmap. Both up and down-regulated p53 targets sorted in descending order by fold change are shown in the heatmap. The values were scaled row-wise. Treatment and genotype assignments are indicated by color bars above the heatmap, and the legend is provided on the bottom right.

In our previous analysis of this dataset using the standard zebrafish transcriptome annotation, we identified 98 known zebrafish orthologs of human p53 target genes as significantly regulated [1]. To demonstrate how much this improved zebrafish transcriptome annotation enhances identification of these zebrafish orthologous p53 targets genes, we performed the same procedure as for IRGs described above. This process found 131 significant p53 target genes (46 up-regulated and 85 down-regulated) (Fig. 4), making up 36.6 % of all potential p53 target genes in zebrafish detectable in the dataset (358 genes) (Supplementary Data; “p53 target genes” sheet). There is some overlap between the IRGs and identified p53 target genes: *fosl1a, atf3, il1b, ccng1, mhc1zba, serpine1*. A recent study of oncogene-induced senescence in zebrafish identified a genetic signature of senescence: up-regulation of *cdkn2a/b, il1b, il6, il11* and *cxcl8a* [18]. This signature overlaps a tissue-ubiquitous senescence signature derived from a meta-analysis of multiple human and mouse datasets: *CDKN1A, CDKN2A, CDKN2B* and *IL6* [19]. Our dataset exhibits up-regulation of most of these markers: *cdkn1a*, cdkn2a/b, *il1b* and *il11* (an IL6-related cytokine), suggesting senescence activation, which is further supported by enrichment of multiple cell-cycle related terms (Supplementary Data, “UP Genes - GO BP and KEGG” sheet).

## 4 Experimental Design, Materials and Methods

### 4.1 Zebrafish husbandry and maintenance

Zebrafish experiments and husbandry followed standard protocols [20]. The ethics approval for the work is described in the Ethics statement. Zebrafish embryos were maintained at 28.5 °C during development and as adults. Embryos were grown in 1x E3 medium.

### 4.2 Drug treatments

Camptothecin (MilliporeSigma, C9911) (CPT) treatments were performed by adding either DMSO (control groups) or the 2 mM stock CPT solution in DMSO to fish water to achieve the indicated concentration and incubating embryos for 4 h. The treated and control embryos were anesthetized and collected for RNA extraction.

### 4.3 RNA sequencing (RNA-Seq) procedures and data analysis

Total RNA samples were measured and analyzed for integrity on Agilent Tape Station. Samples with RNA integration number ≥8 were selected for library preparation. Poly-A enrichment was performed from 20 µg of total RNA to enrich for mRNA (Dynabeads mRNA DIRECT Micro Kit, Thermo Fisher). Poly-A enriched RNA (100 ng) was fragmented using RNase III and purified using magnetic bead clean up module (RNA Seq V2 kit, Life Technologies). The size distribution of the fragmented RNA was assessed on Agilent Tape Station using RNA HS screen tape assay and 50 ng of fragmented polyA-enriched RNA was used to prepare whole transcriptome library (RNA seq V2). Yield and size distribution of the library was analyzed on Agilent Tape Station using D1000 screen tape. Barcoded library was equally pooled and amplified onto Ion Sphere™ Particles (ISPs) from Ion Pi HiQ OT2 kit (Life Technologies). ISPs enriched with template library were loaded onto Ion PI chip V3 and sequenced on Ion Proton from Thermo Fisher. The raw reads had the adapters removed and filtered by the quality of 20 with the Trim Galore (v0.4.4) (Krueger F, https://github.com/FelixKrueger/TrimGalore). The RNA-Seq data were mapped by a two-step STAR (v2.7) [21] mapping using the Genome Reference Consortium Zebrafish Build 11 (danRer11) and the Lawson Lab Zebrafish transcriptome annotation [14] followed by gene abundance quantification using RSEM [22]. Count matrices were evaluated by Principal Component Analysis (PCA) and Multi-dimensional Scaling (MDS) plots and differentially expressed genes were identified using DESeq2 Bioconductor library [23] using a fold-change ≥ 2 threshold and False-discovery rate ≤ 0.05 as parameters. Heatmaps were produced using pheatmap R package (https://cran.r-project.org/web/packages/pheatmap/index.html) after performing variance stabilizing transformation using a function from DESeq2 library. The MA plot was generated using the ggmaplot function from the ggpubr R package (https://cran.r-project.org/web/packages/ggpubr/index.html). In addition, we used a number of standard data science packages for common analytical and visualization tasks: stringr, readr, dplyr, ggsci, ggrepel, forcats and ggplot2. Please see the article repository for further details and code of all the analysis and visualization steps: https://github.com/SergeyPry/CPT_RNA-seq_zebrafish_paper/tree/main.

### 4.4 Bioinformatic tools

Gene Ontology and KEGG pathways enrichment analyses were performed using DAVID [24] (https://david.ncifcrf.gov/summary.jsp) with groups of differentially-expressed genes as a query and the full list of detected genes by RNA-seq in each dataset as a background list. Redundancy among the Gene Ontology Biological Process terms was reduced using REVIGO tool (http://revigo.irb.hr/) [25] with the ‘Medium’ or 0.7 size setting followed by removal of highly dispensable terms.

### 4.5 Data availability

We deposited the RNA-Seq datasets generated as part of this study to Gene Expression Omnibus with the following accession numbers: GSE142440.

## Limitations

Usage of pooled samples for RNA-seq is considered suboptimal because it distorts the inter-individual variation among the sampled animals, but at the time of the study, the technology did not allow for RNA-seq on single embryo samples. The second limitation is that the samples were not from a single clutch of embryos produced by breeding a pair of adult fish with the relevant genotypes. For this experiment, it was not possible to obtain such a group of embryos due to the sample size requirements, stage of the fish, genotyping complexities and, most importantly, the miR-34a maternal contribution, which we aimed to avoid in the sampled animals. Therefore, we cannot exclude that some of the expression changes are due to non-identical genetic backgrounds in the wild-type and *miR-34a-/-* mutant lines despite both lines being derived from the *casper* strain. The response to CPT may also be somewhat different in standard wildtype zebrafish strains (AB, WIK, Tübingen (TU), Tübingen long fin (TL)) to that in *casper*, which means that results from this study may need to be transferred to other strains with caution, but in our experience apoptosis induction is comparable in AB and *casper* strains.

## Ethics Statement

All experiments adhered to ethics policies for the University of Ottawa. Experimental procedures were approved by the University of Ottawa Animal Care Committee (approval number: CHEOe-4166).

## Credit Author Statement

**Sergey V. Prykhozhij:** Conceptualization, Data curation, Formal analysis, Investigation, Methodology, Project administration, Software, Supervision, Validation, Visualization, Writing – original draft, Writing – review & editing. **Kevin Ban:** Investigation, Methodology. **Zane L. Brown:** Investigation. **Kim Kobar:** Investigation, Methodology. **Gabriel Wajnberg:** Data curation, Formal analysis, Software. **Charlotte Fuller:** Investigation, Methodology. **Simi Chacko:** Data curation, Investigation, Resources. **Jacynthe Lacroix:** Data curation, Investigation, Resources. **Nicolas Crapoulet:** Data curation, Resources, Software. **Craig Midgen:** Investigation, Methodology, Resources, Supervision. **Adam Shlien:** Funding acquisition, Resources. **David Malkin:** Conceptualization, Funding acquisition, Project administration, Resources, Writing – review & editing. **Jason N. Berman:** Conceptualization, Funding acquisition, Investigation, Project administration, Resources, Supervision, Writing – original draft, Writing – review & editing.

## Data Availability

Gene Expression OmnibusRNA-seq analysis of gene regulation by miR-34a and expression changes induced by DNA damage in zebrafish (Original data).

## Appendix Supplementary materials

Image, application 1
